# Comorbid physical illnesses in adult outpatients with psychotic disorders: risk factors, psychological functioning, and quality of life outcomes

**DOI:** 10.1007/s00127-021-02034-8

**Published:** 2021-02-22

**Authors:** Wen Lin Teh, Laxman Cetty, Anitha Jeyagurunathan, Fiona Devi, Kumarasan Roystonn, Charmaine Tang, Swapna Verma, Mythily Subramaniam

**Affiliations:** 1 Research Division, Institute of Mental Health, Buangkok Green Medical Park, 10 Buangkok View, Singapore, 539747 Singapore; 2 Department of Early Psychosis Intervention, Institute of Mental Health, Singapore, 539747 Singapore

**Keywords:** Physical comorbidity, Psychotic disorders, Quality of life, Psychological health

## Abstract

**Purpose:**

In contrast to global research, where physical comorbidity in psychotic disorders is established, only a few studies have been conducted in Southeast Asia. With a concerning trend of chronic physical illnesses emerging in adults below the age of 65, an investigation into comorbid chronic physical illnesses in adults diagnosed with psychotic disorders is necessary. This study aims to explore the risk factors, psychological functioning, and quality of life outcomes associated with comorbidity in adults below the age of 65, diagnosed with psychotic disorders, in a multi-ethnic non-Western setting.

**Methods:**

Electronic medical records of 364 patients with psychotic disorders who had provided written consent to participate were screened for co-occurring physical conditions. The majority of participants were female (53.7%), Chinese (69%), single (74.5%), and had tertiary and above education (43%). They were approximately 35 years old on average and the mean age of onset for psychosis was 26.7 years old.

**Results:**

Comorbid physical illnesses were present in approximately a third of adults with psychotic disorders (28%). They typically reported cardiovascular-related diseases, respiratory, and skin conditions. Comorbidity was significantly related to lower physical quality of life. As compared to other types of psychotic disorders, schizophrenia was significantly related to a greater frequency of comorbid physical conditions. Multinomial regression analyses revealed that age, age of onset, Malay and Indian ethnicities were significant factors.

**Conclusion:**

Physical comorbidity in adults below the age of 65 is common, signifying an emerging need to place greater attention into the screening and emphasis on the physical care needs of this age group. Finally, more research is needed to understand the impact of common co-occurring acute and chronic cardiovascular, skin, and respiratory diseases locally.

**Supplementary Information:**

The online version contains supplementary material available at 10.1007/s00127-021-02034-8.

## Introduction

When compared to the general population, individuals with psychotic disorders die 10–15 years prematurely on average [1, 2]. A large proportion of premature mortality is attributed to comorbid medical conditions [1–6] which are common in psychotic disorders. Adapted from Akker et al. [7] and originally from Feinstein et al. [8], comorbidity is defined as any co-occurring chronic condition in addition to the index disease. Depending on the type of medical conditions, physical comorbidity may occur in up to 75% of individuals with psychotic disorders [9, 10]. They are more likely to report diabetes, neurological disorders, metabolic conditions, and respiratory illnesses than the general population [3, 9–14]. Comorbidity is influenced by sociodemographic factors, such as age [15] and gender [9, 16]. While chronic conditions are commonly studied in older adult populations (defined typically in epidemiological studies as ≥ 65 years old) as it is conventionally assumed to be conditions of old age, research has shown that chronic diseases, such as diabetes and cardiovascular diseases, are becoming increasingly common in adults below the age of 65 years [17], and is a concerning trend among adults with severe mental illnesses [18].

Cardiovascular diseases, diabetes, chronic respiratory diseases, and cancer are the four chronic noncommunicable diseases with the highest cause of death and disability in Southeast Asia which is similar to global trends [19]. However, Asian populations have increased morbidity and mortality in important chronic conditions, such as coronary heart disease, as compared to Western populations [20]. Singapore is a Southeast Asian country with approximately four million residents who belong to three major ethnic groups (Chinese, Malay, and Indian). Epidemiological surveys conducted in Singapore found that ethnic minorities in Singapore generally self-report greater physical chronic illnesses [21–23]and while the prevalence of severe mental illnesses in the major ethnic groups are established [24], little is known about physical medical conditions in relation to ethnicity and severe mental illnesses, particularly in psychotic disorders.

In contrast to global research, where physical comorbidity in psychotic disorders is established, only a few studies have been conducted in Southeast Asia. It remains unclear the extent to which global trends concerning comorbid presentations in psychotic disorders are generalizable to and descriptive of a non-western population that is also multi-ethnic. Epidemiological surveys that investigate severe mental illnesses typically do not include questions on psychotic disorders in shorter questionnaires due to the lack of validity of self-report in diagnosing psychotic disorders [3, 25, 26], contributing to the lack of data. Existing local studies that use administrative data are however limited to the study of schizophrenia only [27, 28], whereas other schizophrenia spectrum disorders and mood disorders with psychotic symptoms are largely ignored. Consequently, local research investigating the impact of physical comorbidity on psychosocial outcomes, such as quality of life (QOL) and psychological functioning, among individuals with psychotic disorders is generally lacking. Lastly, while it is typical for studies to include chronic medical conditions since they are more pervasive in older adults [7, 8], such strict inclusion criteria has inevitably contributed to a lack of breadth in terms of the scope of medical conditions investigated. Acute medical conditions, such as bacterial infections (i.e., tuberculosis) remain poorly studied as compared to chronic conditions like diabetes mellitus [12].

Given the present gaps in the current literature, this study aims to explore the types of comorbid physical illnesses, risk factors, psychological and quality of life outcomes of comorbid physical disorders in younger adult patients with psychotic disorders. From past reports on various severe mental illnesses, it is hypothesized that having comorbid medical conditions would be significantly associated with older age and lower quality of life [15, 26, 29, 30]. Supplementary to the study of comorbidity, we aim to also investigate co-occurring acute and chronic medical conditions at a descriptive level and its relation to QOL and psychosocial outcomes, as there is an exploratory value to do so [7].

## Methods

### Procedure

Outpatients with psychotic disorders were recruited from the Institute of Mental Health (IMH), a tertiary psychiatric hospital in Singapore, between January 2018 and April 2019. According to the Diagnostic and Statistical Manual for Mental Disorders (DSM-IV), psychotic disorders refer to a group of illnesses that are characterized by hallucinations, delusions, disorganized speech, grossly disorganized behavior, and negative symptoms [31]. Participants were referred to the research team by their clinicians, who were aware of the full objectives of the research study, or they had approached the research team in the outpatient clinics. Participants were recruited using an information flyer that described the details of the study. Participants’ diagnoses were confirmed using their electronic medical records or by their doctors. Participants who agreed to participate signed written informed consent forms prior to completing a set of questionnaires by paper and pen. Their responses were subsequently entered electronically by the research team to prepare for analyses. The inclusion criteria for the study were: Singapore citizens or Permanent residents, aged 21–65 years old, have a history of a psychotic disorder, and are literate in the English language. The exclusion criteria were substance-induced psychotic disorders (as they have distinct clinical and demographic presentations than primary psychotic disorders [32]), those with intellectual disabilities, and those who were unable to read or speak in English. This study was approved by the relevant institutional review committees—the National Health Group (NHG) Domain Specific Review Board (DSRB) and the IMH Institutional Research Review Committee (IRRC).

### Materials

Medical records of patients who provided written consent to participate in this study were screened. For the purposes of this study, we extracted the primary psychiatric diagnoses, existing acute and chronic medical conditions, and age of onset of psychotic disorders. Participants self-reported their age, marital status, gender, highest education levels, and ethnicity.

### Measures

i. Depression Anxiety Stress Scales (DASS-21) [33]: This is the short version of DASS that measures subscales of Depression, Anxiety, and Stress (seven items per subscale). The DASS-21 scale asks participants to respond the extent to which the statements applied to them over the past week. Likert responses range from 0 (never) to 3 (almost always). This is a valid measure for use on clinical and non-clinical samples [34]. Internal reliability of the subscales in the present sample was excellent, ranging from 0.83 to 0.90.
ii. World Health Organization—Quality of Life (WHOQOL-BREF) [35]: This questionnaire measures four domains of quality of life: physical health, psychological health, social relationships, and environment. This scale had been found to a valid measure for QOL in psychiatric outpatients including psychotic disorders [36]. Cronbach alpha ranged from acceptable (0.6 for the physical component) to excellent (0.8 for the environment component) in this study.

### Physical medical conditions

As a large number of medical conditions were evaluated, we grouped individual (chronic and acute) conditions into 15 categories that represented the 10th version of the International Statistical Classification of Diseases and Related Health Problems (ICD-10). Classifying individual medical conditions into ICD-10 medical categories ensured adequate parsimony and sufficient statistical power for analyses involving rarer medical conditions [37]. The 15 categories were: neoplasm (e.g., mixed glioma, carcinoma, etc.), infectious or parasitic diseases (e.g., tuberculosis, viral warts, varicella, etc.), disorders of the circulatory system (e.g., myocardial infarction, stroke, etc.), skin (e.g., dermatitis, alopecia, etc.), musculoskeletal system and connective tissue (e.g., scoliosis, arthropathy, back pain, etc.), digestive (e.g., enteritis, irritable bowel syndrome, etc.), respiratory (e.g., asthma, rhinitis, etc.), ear/mastoid (e.g., tinnitus, otitis media, etc.), adnexa/eye (e.g., conjunctivitis, myopia, etc.), nervous system (e.g., epilepsy, migraine, tardive dyskinesia, etc.), blood (e.g., anemia, etc.), endocrine/metabolism/nutritional (e.g., hyperlipidemia, obesity, diabetes mellitus, etc.) and genitourinary tract (e.g., bladder stone, amenorrhea, etc.).

To study comorbidity, a subset of these conditions were subsequently re-categorized into nine categories representing fifteen chronic conditions from the composite international diagnostic interview (CIDI) checklist [26]. These categories were typically used in local population-based epidemiological studies as they were locally relevant and selected based on the advice by various stakeholders [38]. They were: respiratory conditions (asthma, emphysema), diabetes or hyperglycemia, hypertension, hyperlipidemia, chronic pain (arthritis, rheumatism, migraine, back problems), cancer, neurological disorders (epilepsy, Parkinson’s disease), cardiovascular diseases (stroke, heart attack, angina, or other heart diseases), ulcer and chronic inflamed bowel (stomach ulcer, enteritis, colitis). Comorbidity was operationalized as having at least one comorbid chronic condition. A comorbid condition thus refers to a co-occurring chronic medical/physical condition in addition to the index condition of psychotic disorders.

### Statistical analyses

The initial calculation of descriptive statistics, such as the means and standard deviations, were conducted to understand the distribution of the sample’s characteristics. Analysis of Variance (ANOVA) and test of independence chi-square tests were conducted to ascertain if there were significant differences/associations between the three groups and sociodemographic variables. Phi coefficients were calculated to determine the correlation between physical categories which had dichotomous indicators (Yes/No). Non-parametric Kruskal–Wallis tests were utilized to determine if there were significance differences in the frequency of general medical and comorbid conditions between three psychotic disorder groups. To investigate the risk factors of comorbidity, multinomial logistic regression was conducted using three outcomes: none, one, and two or more comorbid conditions. Finally, multiple linear regression analyses were conducted to ascertain if there were significant associations between comorbidity and anxiety, depression, or stress levels (as measured by DASS-21), and four dimensions of quality of life such as physical health, psychological health, social relationships, and environment QOL (as measured by WHOQOL-BREF), while accounting for sociodemographic covariates.

To explore the impact of co-occurring ICD-10 general medical conditions (pertaining to all physical conditions regardless of chronicity) in individuals with psychotic disorders, separate descriptive statistics, ANOVA, the calculation of phi coefficients, and multiple linear regression analyses were conducted. All tests utilized a 0.05 criterion for statistical significance and were conducted in IBM SPSS Statistics for Windows, version 23. The results are presented as supplementary material of this manuscript. Missing values were deleted listwise.

## Results

### Sample characteristics

Data of 364 participants with psychotic disorders were utilized for this study. Participants were approximately 35 years old on average (ranging from 21 to 65 years old), and the mean age of onset was 26.7 years. One participant was missing a data point on diagnosis, and hence the analyses involving comparison between psychotic disorder groups were based on 363 participants. To be consistent with the classification of psychotic disorders in the Diagnostic and Statistical Manual of Mental Disorders (DSM-5), individuals with schizophrenia, schizoaffective disorder, schizophreniform disorder, brief psychotic disorder, delusional disorder, and psychotic disorder NOS were initially classified as non-affective psychotic disorders. However, as preliminary analyses found that the other non-affective psychotic disorder group (age: *M* = 32.3, *SD* = 9.5) was significantly younger than those with schizophrenia (age: *M* = 37.2, *SD* = 11), and as initial chi-square analyses suggested that individuals with schizophrenia had greater comorbid conditions, it was decided to separate individuals with schizophrenia from the rest of the non-affective psychotic disorder as a separate group. Individuals with either Bipolar I disorder or Major Depressive Disorder and with psychotic features were classified as having affective psychotic disorders (age: *M* = 27, *SD* = 7.6). Age of onset was not significantly different between the three psychotic disorder categories. Majority of participants were female (53.7%), Chinese (69%), single (74.5%), and had tertiary and above education (43%). There were significant differences in only one domain of QOL (social relationships) between the psychotic disorder groups. Post hoc pair-wise comparisons using Bonferroni corrections revealed a marginal significant difference between schizophrenia and other non-affective psychotic disorder groups (*p* = 0.05). The results are summarized in Table 1.

**Table 1:** Sample characteristics, DASS-21, and WHOQOL-BREF scores among psychotic disorder groups

	All	Schizophrenia	Other non-affective psychotic disorders	Affective psychotic disorders	
	(*n* = 364)	(*n* = 231)	(*n* = 109)	(*n* = 23)	
Mean (sd)					*F (df*^*b*^*, df*^*w*^*)*
Age	35.2 (10.8)	37.2 (11)	32.3 (9.5)	27 (7.6)	**16.0 (2, 357)**
Age of onset	26.7 (7.93)	26.8 (7.8)	26.9 (8.5)	23.9 (4.2)	1.48 (2, 356)
DASS-21 scores					
Anxiety	10.2 (8.8)	10.1 (8.7)	9.9 (8.7)	12 (10.6)	0.53 (2, 358)
Depression	11.1 (10.3)	11.4 (10)	10 (10.1)	14.4 (12.7)	1.95 (2, 358)
Stress	11.4 (10)	11.7 (10)	10.2 (9.5)	14.7 (11.3)	2.18 (2, 357)
WHOQOL-BREF scores					
Physical health	14.3 (2.5)	14.1 (2.6)	14.5 (2.5)	14.6 (2)	1.06 (2, 357)
Psychological health	12.9 (3.1)	12.8 (3.2)	13.3 (2.8)	12.4 (2.9)	1.17 (2, 359)
Social relationships	13.2 (3.2)	12.9 (3.3)	13.8 (2.7)	12.4 (3.2)	**3.41 (2, 327)**
Environment	13.7 (2.8)	13.5 (2.9)	14 (2.4)	14.3 (2.6)	1.98 (2, 358)

*n* (%)					χ^2^
Gender					*ns*
Female	196 (53.7)	124 (34.2)	55 (15.2)	16 (4.4)	
Male	168 (46.0)	107 (29.5)	54 (14.9)	7 (1.9)	
Ethnicity					*ns*
Chinese	252 (69.0)	157 (43.3)	76 (20.9)	19 (5.2)	
Malay	57 (15.6)	39 (10.7)	15 (4.1)	2 (0.6)	
Indian	40 (11.0)	26 (7.2)	12 (3.3)	2 (0.6)	
Others	15 (4.1)	9 (2.5)	6 (1.7)	0 (0)	
Marital status					*ns*
Single	272 (74.5)	176 (48.5)	82 (22.6)	14 (3.9)	
In a relationship/Married	71 (19.5)	38 (10.5)	24 (6.6)	8 (2.2)	
Divorced/separated/ widowed	21 (5.8)	17 (4.7)	3 (0.8)	1 (0.3)	
Highest education					**9.8**
Secondary and below	102 (27.9)	73 (20.1)	26 (7.2)	2 (0.6)	
Pre-tertiary	105 (28.8)	112 (30.9)	47 (12.9)	11 (3)	
Tertiary and above	157 (43.0)	46 (12.7)	36 (9.9)	10 (2.8)	

*F* and χ2 (Chi-square) statistics are based on Analysis of Variance (ANOVA) and test of independence chi-square tests; values in bold indicate significance at *p* < .05; *ns* indicate not significant. *df*^b^, *df*^w^ refer to between and within degrees of freedom, respectively

### Comorbidity between groups

Almost a third (28%) of participants with psychotic disorders had at least one comorbid condition. 17% of participants had one comorbid condition only whereas 11% of participants had two or more comorbid conditions. Hyperlipidemia was the most prevalent comorbid condition and approximately 14% of participants with psychotic disorders had the condition. This was followed by diabetes or hyperglycemia (7.4%), chronic pain (7.1%), respiratory conditions (6.1%), and others. When considering all general medical conditions (i.e., regardless of chronicity), approximately half (49.3%) of all participants had at least one condition. Majority had respiratory or skin conditions (25.5%).

As the statistical distribution of the frequency count of comorbid conditions appeared positively skewed (i.e., most participants had none as compared to six comorbid conditions), we performed non-parametric Kruskal–Wallis tests to determine if there were significant differences in the frequency count of comorbid conditions between the three psychotic disorder groups. Significant differences were present, *χ*^2^(2) = 9.72, *p* = 0.008, among the three groups. Post hoc pairwise comparisons using Kruskal–Wallis tests revealed that there were significant differences between schizophrenia and non-affective psychotic disorder groups, *χ*^2^(1) = 6.63, *p* = 0.01, and between schizophrenia and affective psychotic disorder groups, *χ*^2^(1) = 4.07, *p* = 0.044. There were no significant differences between other non-affective and affective psychotic disorder groups. These results are summarized in Table 2. No significant differences were recorded for general medical conditions (supplementary Table 1).

**Table 2:** Chi-square and Phi coefficient statistics between psychotic disorder groups and comorbid medical categories (*n* = 364)

		All (*n* = 364)	(a) Schizophrenia (*n* = 231)	(b) Non-affective psychotic disorders (*n* = 109)	(c) Affective psychotic disorders (*n* = 23)	χ^2^								
	No. of comorbid conditions	*n* (%)	*n* (%)	*n* (%)	*n* (%)	(a) vs (b)	(a) vs (c)	(b) vs (c)						
	None	261 (71.5)	155 (42.7)	85 (23.4)	20 (5.5)	**6.63**	**4.07**	0.829						
	One	63 (17.3)	47 (12.9)	15 (4.1)	1 (0.3)									
	Two or more	40 (11.0)	29 (8)	9 (2.5)	2 (0.6)									
	*One or more*	103 (28.2)	76 (20.9)	24 (6.6)	3 (0.8)									

						Phi coefficients								
	Medical categories					1	2	3	4	5	6	7	8	9
1	Respiratory	23 (6.1)	12 (3.3)	9 (2.5)	1 (0.3)	1								
2	Cardiovascular	6 (1.6)	4 (1.1)	1 (0.3)	1 (0.3)	**0.23**	1							
3	Hypertension	15 (3.9)	11 (3.0)	2 (0.6)	1 (0.3)	**0.12**	0.08	1						
4	Diabetes or Hyperglycemia	27 (7.4)	22 (6.1)	5 (1.4)	0 (0)	0.06	**0.13**	**0.46**	1					
5	Hyperlipidemia	53 (13.9)	41 (11.3)	8 (2.2)	2 (0.6)	**0.12**	0.07	**0.31**	**0.44**	1				
6	Cancer	1 (0.3)	1 (0.3)	0 (0)	0 (0)	− 0.01	− 0.01	− 0.01	− 0.02	**0.13**	1			
7	Neurological diseases	4 (1.1)	3 (0.8)	1 (0.3)	0 (0)	− 0.03	− 0.01	− 0.02	0.07	− 0.01	− 0.01	1		
8	Chronic Pain	27 (7.1)	18 (5)	8 (2.2)	1 (0.3)	− 0.03	0.05	0.00	0.04	− 0.01	− 0.01	**0.17**	1	
9	Ulcers and Chronic inflamed bowel	10 (2.6)	8 (2.2)	1 (0.3)	0 (0)	**0.17**	− 0.02	− 0.03	0.08	− 0.01	− 0.01	**0.14**	0.08	1

χ^2^ values are based on pairwise comparisons using Kruskal–Wallis non-parametric tests between the number of comorbid conditions (none, one, two or more comorbid conditions) and psychotic disorder categories (schizophrenia, non-affective, affective psychotic disorder groups); phi coefficients represent correlations between two comorbid physical categories (Yes/No); values in bold indicate significance at *p* < .05

### Associations of comorbidity

Multinomial logistic regression revealed that older age, earlier age of onset, Malay and Indian ethnic groups (as compared to Chinese ethnic group) were risk factors associated with greater odds of having one, and two or more comorbid conditions as compared to none. Marital status, education levels, and the type of psychotic diagnosis were not significantly associated with comorbidity (Table 3).

**Table 3:** Risk factors of comorbidity

				Multinomial Regression Analyses					
	None^	One^	Two or more^	One vs None			Two vs None		
	*n* (%)	*n* (%)	*n* (%)	OR	95% CI	*P* value	OR	95% CI	*P* value
Age (Mean (SE))	33.2 (0.6)	37.8 (1.5)	42.9 (1.8)	**1.08**	1.042, 1.121	< .001	**1.12**	1.076, 1.168	< .001
Age of Onset (Mean (SE))	26.5 (0.5)	26.5 (1.1)	27.7 (1.4)	**0.94**	.898, 0.987	0.013	**0.94**	.891, 0.989	0.018
Sex									
Female	146 (40.1)	32 (8.8)	18 (4.9)	0.78	0.427, 1.445	0.437	0.61	0.285, 1.305	0.202
Male	115 (31.6)	31 (8.5)	22 (6)	1					
Ethnicity									
Chinese	192 (52.7)	37 (10.2)	23 (6.3)	1					
Malay	34 (9.3)	14 (3.8)	9 (2.5)	**3.55**	1.568, 8.048	0.002	**3.41**	1.248, 9.336	0.017
Indian	22 (6)	11 (3)	7 (1.9)	**3.45**	1.462, 8.150	0.005	**3.32**	1.134, 9.701	0.029
Others	13 (3.6)	1 (0.3)	1 (0.3)	0.50	0.060, 4.149	0.519	0.77	0.084, 7.095	0.820
Marital									
Single	199 (54.7)	46 (12.6)	27 (7.4)	1					
In a relationship/married	47 (12.9)	14 (3.8)	10 (2.7)	1.15	0.516, 2.548	0.737	1.20	0.457, 3.086	0.724
Divorced/separated/widowed	15 (4.1)	3 (0.8)	3 (0.8)	0.39	0.076, 2.027	0.264	0.76	0.165, 3.532	0.731
Education									
Secondary and below	71 (19.5)	17 (4.7)	14 (3.8)	1					
Pre-tertiary	71 (19.5)	20 (5.5)	14 (3.8)	1.79	0.788, 4.057	0.164	1.73	0.669, 4.468	0.259
Tertiary and above	119 (32.7)	26 (7.1)	12 (3.3)	1.70	0.770, 3.769	0.188	1.11	0.423, 2.903	0.835
Psychiatric diagnoses									
Schizophrenia	155 (42.7)	47 (12.9)	29 (8)	1					
Other non-affective psychotic disorders	85 (23.4)	15 (4.1)	9 (2.5)	0.80	0.399, 1.603	0.528	1.01	0.421, 2.437	0.977
Affective psychotic disorders	20 (5.5)	1 (0.3)	2 (0.6)	0.29	0.034, 2.389	0.248	1.40	0.230, 8.502	0.716

^Outcome variables are none, one comorbid, two or more comorbid conditions; OR is odds ratio; 95% CI is 95% confidence intervals; values in bold indicate significance at *p* < .05

Multivariate linear regression analyses revealed that comorbidity was not associated with the majority of psychological and QOL (except for physical health QOL) domains after accounting for sociodemographic information, psychotic disorder type, and age of onset of psychosis. Having one comorbid condition (as compared to none) was significantly and negatively associated with physical health and social relationships QOL scores (Table 4). Having at least one co-occurring medical condition (as compared to none) was associated with higher levels of anxiety, depression, stress DASS-21 scores, and lower physical health QOL scores. Having at least two medical conditions (as compared to none), was significantly associated with lower physical health QOL scores only (supplementary Table 2).

**Table 4:** Multiple linear regression analyses of comorbidity, DASS-21, and WHOQOL-BREF scores

Chronic conditions	Quality of life (WHOQOL-BREF)							
	Physical Health		Psychological Health		Social Relationships		Environment	
	*B (SE)*	95% CI	*B (SE)*	95% CI	*B (SE)*	95% CI	*B (SE)*	95% CI
One or more	**− 0.7 (0.3)**	(− 1.33 − 0.02)	− 0.3 (0.4)	(− 1.05, 0.51)	− 0.8 (0.4)	(− 1.59, 0.05)	0.1 (0.3)	(− 0.61, 0.80)
One	**− 0.9 (0.4)**	(− 1.62 − 0.09)	− 0.6 (0.5)	(− 1.46, 0.34)	**− 1.0 (0.5)**	(− 1.93, − 0.03)	− 0.2 (0.4)	(− 0.44, 1.56)
Two or more	− 0.4 (0.5)	(− 1.30, 0.54)	0.2 (0.6)	(− 0.90, 1.32)	− 0.4 (0.6)	(− 1.57, 0.76)	0.6 (0.5)	(− 1.00, 0.64)
vs. None	Ref.		Ref.		Ref.		Ref.	Ref.

Chronic conditions	Psychological outcome measures (DASS 21)					
	Anxiety		Depression		Stress	
	*B (SE)*	95% CI	*B (SE)*	95% CI	*B (SE)*	95% CI
*One or more*	0.4 (1.1)	(− 1.74 2.60)	1.6 (1.3)	(− 0.96, 4.15)	1.7 (1.2)	(− 0.79, 4.17)
One	0.1 (1.3)	(− 2.47 2.57)	2.0 (1.5)	(− 0.95, 4.99)	1.9 (1.5)	(− 1.01, 4.73)
Two or more	1.1 (1.6)	(− 2.00 4.16)	0.9 (1.8)	(− 2.76, 4.50)	1.4 (1.8)	(− 2.12 4.91)
vs. None	Ref.		Ref.		Ref.	

Analyses involved regressing quality of life domains or psychological outcomes on the number of comorbid conditions (one or more vs. none; one, two or more, vs. none) while controlling for sociodemographic information such as gender, ethnicity, marital status, highest education levels, age, age of onset, and psychotic disorder categories; values in bold indicate significance at *p* < .05

Results on co-occurring general medical conditions are summarized in the supplementary section.

## Discussion

Thirty percent of individuals with psychotic disorders had at least one comorbid physical condition. These results were lower than those reported elsewhere among individuals with psychotic disorders or with other forms of severe mental illnesses [9, 13, 16, 26], with an exception of one study which found comorbidity in approximately 22% of outpatients with schizophrenia which was lower than the present figures [27]. Given the influence of age on comorbidity, the general lack of agreement between past and present findings were expected since the present sample population consisted of younger adults (i.e., the average age of the current sample was 35 years old). Furthermore, the lack of agreement could be explained by differences in methodology (i.e., the use of either self-report vs. medical records).

Regardless of the disparity in the proportions however, the trend of comorbidity remained largely the same for all non-affective psychotic disorders. Certain comorbid conditions, such as chronic pain, hypertension, diabetes, hyperlipidemia, and respiratory conditions were more common, which corroborated past findings [9, 16, 39]. These results may be explained by a multitude of underlying factors, such as genetics, lifestyle risk factors, and physio-pathological vulnerabilities that are expressed in those presenting with psychotic disorders [40–44]. Unhealthy lifestyle habits, such as smoking, poor diet, and sedentary behaviors too may contribute to the comorbid presentations of cardiovascular and metabolic-related disorders [45, 46]. Comparisons between the three psychotic disorder groups revealed that individuals with schizophrenia had significantly greater frequency of comorbid conditions. This was expected as schizophrenia, relative to other non-affective or affective psychotic disorders, is more debilitating, such as having more pronounced (negative) symptoms [47], which may be positively linked to co-occurring physical health complications [48, 49]. However, due to the small numbers included in the affective psychotic disorder group, results pertaining to this group should be interpreted with caution. A recent report had found that cardiovascular, neurological, or skin-related diseases, each combined with respiratory diseases, had the highest impact on mortality rates [50]. As respiratory and skin conditions were reported in a sizable proportion of individuals, and as respiratory and skin conditions were significantly correlated locally, our findings, together with other evidences found overseas, would warrant a thorough examination of local data of these combinations in psychotic disorders.

Subsequent multinomial regression analyses revealed that the psychotic disorder type was not significantly associated with comorbidity after controlling for covariates, suggesting that other risk factors (i.e., age, age of onset, Malay and Indian ethnicities) played a more significant role. Age has been consistently documented to have a significant and important impact on comorbidity [15, 25, 26]. According to biomarker research, aging is accelerated in individuals with schizophrenia due to oxidative stress and inflammation, contributed by an interplay of unhealthy lifestyle habits and physiological abnormalities [51]. Moreover, a significant effect of an early age of onset could indicate that the vulnerabilities associated with those previously described physio-pathological links, such as changes to immune system activity or unhealthy lifestyle behaviors found in individuals with schizophrenia, could be present at a young age thus making comorbidity more likely with age [52, 53].

Malay and Indian ethnic groups were at least three times more likely to have comorbid conditions. Our findings were expected as past research had shown greater prevalence of self-reported chronic conditions, such as diabetes, high cholesterol, and heart diseases, stroke, in Malay and/or Indian ethnic groups [21, 23, 54]. Various cardiovascular related complications, such as central arterial stiffness [55] and glycaemic control [56], have been found to be associated with Malay and/or Indian ethnic groups diagnosed with diabetes for instance, suggesting that lifestyle risk factors, such as diet, could have also contributed to the increased likelihood of certain comorbid presentations in these ethnic groups [22].

Being an ethnic minority could potentially compound the odds of receiving worse health outcomes over and above the aforementioned heightened risk of morbidity and mortality associated with comorbidity. This is due to known ethnic disparities associated with a myriad of factors, such as the association with social economic status (SES) [57]. Ethnic minorities could face increased barriers to primary care, experience lower health service engagement, all potentially leading to poorer health outcomes [58]. While our results imply a need for more awareness and sensitivity over these presentations among Malay and Indian adult patients with psychotic disorders, addressing financial concerns of low SES, could further uplift disparities and improve barriers to healthcare and health outcomes [57].

Adult individuals presenting with at least one co-occurring ICD-10 medical condition were more likely to self-rate higher anxiety, depression, stress levels, and lower physical health QOL than those without these presentations. When considering comorbid conditions only however, only physical health QOL tended to be negatively self-rated but not the psychological outcomes measured by DASS-21, which corroborated baseline findings from a past report [27]. In addition, when compared to those without comorbidity, individuals with one comorbid condition but not two or more comorbid conditions, were significantly associated with lower social relationships QOL and physical health QOL. To ascertain the reasons, additional post hoc descriptive and chi-square analyses were conducted. As compared to individuals with two or more comorbid conditions who had significantly greater proportions of those with comorbid respiratory, hypertension, diabetes/hyperglycemia, or hyperlipidemia conditions, individuals with one comorbid condition only had greater proportions of those with comorbid chronic pain (one vs two or more: 18.4% vs 7.8% with chronic pain conditions, though not significant). Thus, the association between physical health QOL and one comorbid condition only could be explained by a greater presence of comorbid chronic pain. The inverse relationship that was significant between having one comorbid condition only and social relationships domain of QOL could be explained by the fact that pain symptoms had been found to be temporally associated with a lack of a supportive social network [59, 60].

Similarly, post hoc descriptive analyses were conducted to explain the associations between QOL and ICD-10 medical conditions. Relative to those with one medical condition, individuals with two or more medical conditions who had significantly greater proportions of most conditions such as disorders of the circulatory system, nervous system, musculoskeletal, and digestive systems, could have experienced a lowered capacity to perform daily functions (i.e., a lack of energy, or needing medication to daily life functions) and therefore reported lower physical health QOL.

As a whole, the results gathered could be clarified by the fact that different medical conditions impact QOL and psychological health in many different ways. Since we analyzed a summation of disease indicators as opposed to single indicators (as the former is more parsimonious), it had inevitably created limits in the ways QOL or psychological outcomes could be analyzed. An additional limitation is that all medical conditions were treated as equally severe and the analyses were carried out without introducing weights to discern conditions by severity. Therefore, the results of our study are only preliminary and future research could expand the understanding of these relationships by addressing the severity and impacts of each comorbid condition on QOL or psychological outcomes. While this study did not take into account the severity of psychotic symptoms, it is expected that those who experience psychotic symptoms will self-rate higher psychological distress and lower QOL.

Other caveats concerning sample characteristics include the fact that the majority of participants had tertiary education. Thus, our study sample may not be sufficiently representative of the population of adults experiencing psychotic disorders due to possible selection bias.

To our knowledge, this study is among the few to investigate physical comorbidity in a large diverse sample of outpatients with psychotic disorders in a tertiary psychiatric care institution in a multi-ethnic non-western setting. Approximately three of ten individuals had at least one comorbid condition. While having at least one comorbid condition was significantly associated with lower quality of life in terms of physical health, the patterns of association between the different domains of QOL and comorbidity may vary depending on the types of medical condition. The majority of those with psychotic disorders reported co-morbid cardiovascular-related diseases. Skin and respiratory conditions were also found to co-occur in a sizable proportion of these individuals, warranting further research in this area. It was also found that individuals with schizophrenia had a significantly greater number of comorbid conditions as compared to other non-affective and affective psychotic disorders. However, additional analyses revealed that psychotic disorder type was not a significant predictor of comorbidity. Instead, older age, younger age of onset of psychotic disorders, Malay and Indian ethnic groups were stronger risk factors. Due to the nature of the study, the results of the study are preliminary and require further research to confirm the results.

## Supplementary Information

Below is the link to the electronic supplementary material.

Supplementary file1 (DOCX 23 KB)

Supplementary file2 (DOCX 16 KB)
