# Supplementary material for: Comorbid physical illnesses in adult outpatients with psychotic disorders: risk factors, psychological functioning, and quality of life outcomes
Source: Soc Psychiatry Psychiatr Epidemiol. 2021 Feb 22;56(9):1633–43. doi: 10.1007/s00127-021-02034-8 (PMC8429359; doi:10.1007/s00127-021-02034-8)
Supplement: Supplementary file 1 — Supplementary file1 (DOCX 23 KB) [file 127_2021_2034_MOESM1_ESM.docx]

| Supplementary Table I: Chi-square and Phi coefficient statistics between psychotic disorder groups and all co-occurring medical categories (n=364) | | | | | | | | | | | | | | | | | | |  |
| --- | --- | --- | --- | --- | --- | --- | --- | --- | --- | --- | --- | --- | --- | --- | --- | --- | --- | --- | --- |
|  |  | All  (n=364) | (a) Schizophrenia  (n=231) | (b) Non-affective psychotic disorder (n=109) | (c) Affective psychotic disorder  (n=23) | chi-square^ | | |  |  |  |  |  |  |  |  |  |  | |
| Physical conditions | | n (%) | n (%) | n (%) | n (%) | (a) vs (b) | (a) vs (c) | (b) vs (c) |  |  |  |  |  |  |  |  |  |  | |
|  | None | 184 (50.4) | 116 (32) | 57 (15.75) | 11 (3) | 0.130 | 0.010 | 0.080 |  |  |  |  |  |  |  |  |  |  | |
|  | One | 98 (26.8) | 61 (16.8) | 29 (8) | 8 (2.2) |  |  |  |  |  |  |  |  |  |  |  |  |  | |
|  | Two or more | 82 (22.5) | 54 (14.9) | 23 (6.3) | 4 (1.1) |  |  |  |  |  |  |  |  |  |  |  |  |  | |
|  | *One or more* | 180 (49.3) | 115 (31.7) | 52 (14.3) | 12 (3.3) |  |  |  |  |  |  |  |  |  |  |  |  |  | |
|  |  |  |  |  |  | Phi coefficients | |  |  |  |  |  |  |  |  |  |  |  | |
| Physical categories | |  |  |  |  | 1 | 2 | 3 | 4 | 5 | 6 | 7 | 8 | 9 | 10 | 11 | 12 | 13 | |
| 1 | Neoplasm | 4 (1.1) | 2 (0.6) | 1 (0.3) | 1 (0.3) | 1 |  |  |  |  |  |  |  |  |  |  |  |  | |
| 2 | Infectious diseases | 9 (2.5) | 5 (1.4) | 3 (0.8) | 0 (0) | -0.02 | 1 |  |  |  |  |  |  |  |  |  |  |  | |
| 3 | Circulatory System | 19 (5.2) | 14 (3.9) | 3 (0.8) | 2 (0.6) | 0.09 | -0.04 | 1 |  |  |  |  |  |  |  |  |  |  | |
| 4 | Skin | 46 (12.6) | 24 (6.6) | 19 (5.2) | 3 (0.8) | 0.04 | **0.21** | 0.06 | 1 |  |  |  |  |  |  |  |  |  | |
| 5 | Musculoskeletal | 28 (7.7) | 19 (5.2) | 8 (2.2) | 1 (0.3) | 0.07 | 0.02 | 0.03 | **0.11** | 1 |  |  |  |  |  |  |  |  | |
| 6 | Digestive | 24 (6.6) | 17 (4.7) | 5 (1.4) | 2 (0.6) | -0.03 | 0.10 | -0.06 | **0.23** | **0.21** | 1 |  |  |  |  |  |  |  | |
| 7 | Respiratory | 47 (12.9) | 26 (7.2) | 16 (4.4) | 4 (1.1) | -0.04 | 0.10 | **0.17** | **0.22** | **0.10** | **0.23** | 1 |  |  |  |  |  |  | |
| 8 | Ear/Mastoid | 8 (2.2) | 3 (0.8) | 4 (1.1) | 1 (0.3) | -0.02 | -0.02 | -0.04 | 0.06 | 0.03 | **0.11** | 0.05 | 1 |  |  |  |  |  | |
| 9 | Adnexa/Eye | 10 (2.7) | 8 (2.2) | 2 (0.6) | 0 (0) | -0.02 | 0.08 | -0.04 | **0.19** | **0.14** | **0.16** | 0.09 | -0.03 | 1 |  |  |  |  | |
| 10 | Nervous System | 25 (6.9) | 15 (4.1) | 8 (2.2) | 2 (0.6) | 0.08 | -0.04 | 0.08 | 0.06 | **0.13** | 0.06 | 0.03 | 0.03 | **0.15** | 1 |  |  |  | |
| 11 | Blood | 12 (3.3) | 6 (1.7) | 5 (1.4) | 1 (0.3) | -0.02 | -0.03 | 0.10 | 0.02 | -0.05 | 0.01 | 0.02 | -0.03 | -0.03 | 0.07 | 1 |  |  | |
| 12 | Endocrine/Nutri/Metabolism | 74 (20.3) | 56 (15.4) | 15 (4.1) | 3 (0.8) | 0.08 | -0.04 | **0.40** | 0.01 | 0.09 | 0.09 | **0.11** | **0.11** | -0.04 | 0.03 | 0.10 | 1 |  | |
| 13 | Genitourinary | 22 (6) | 13 (3.6) | 6 (1.7) | 3 (0.8) | 0.08 | 0.03 | **0.15** | 0.08 | **0.19** | **0.12** | **0.12** | -0.04 | -0.04 | 0.07 | -0.05 | 0.07 | 1 | |
| ICD10 is 10th version of the International Statistical Classification of Diseases and Related Health Problems; chi-square^ based on Kruskal-Wallis non-parametric test; phi coefficients represent correlations between two physical categories (Yes/No) at a time; bold values indicate significant at *p<*.05. | | | | | | | | | | | | | | | | | | |  |

| Supplementary Table II: Multiple linear regression analyses of all ICD10 medical conditions, DASS21, and WHOQOL- BREF scores. | | | | | | | | |
| --- | --- | --- | --- | --- | --- | --- | --- | --- |
|  | QUALITY OF LIFE (WHO-QOL BREF) | | | | | | | |
|  | Physical Health | | Psychological Health | | Social Relationships | | Environment | |
| ICD10 medical conditions | *B (SE)* | 95% CI | *B (SE)* | 95% CI | *B (SE)* | 95% CI | *B (SE)* | 95% CI |
| *One or more* | **-0.72 (0.28)** | (-1.27, -.17) | -0.22 (0.34) | (-.887 .443) | -0.53 (.36) | (-1.243, .179) | -.14 (0.3) | (-.737, .459) |
| One | -0.60 (0.33) | (-1.24, .040) | 0.045 (0.40) | (-0.73, 0.82) | -0.35 (0.42) | (-1.19, 0.48) | -0.02 (0.3) | (-0.72, 0.69) |
| Two or more | **-0.88 (0.36)** | (-1.58 -.180) | -0.57 (0.43) | (-1.42, 0.28) | -0.76 (0.46) | (-1.66, 0.14) | -0.30 (0.4) | (-1.07, 0.47) |
| vs. None | ref |  | ref |  | ref |  | ref |  |
|  | PSYCHOLOGICAL OUTCOME MEASURES (DASS 21) | | | | | |  |  |
|  | Anxiety | | Depression | | Stress | |  |  |
| ICD10 medical conditions | *B (SE)* | 95% CI | *B (SE)* | 95% CI | *B (SE)* | 95% CI |  |  |
| *One or more* | **2.5 (0.9)** | (0.63, 4.27) | **2.7 (1.1)** | (0.56, 4.87) | **2.61 (1.10)** | (.53, 4.70) |  |  |
| One | **3.3 (1.1)** | (1.19, 5.46) | **3.8 (1.3)** | (1.33, 6.36) | **2.99 (1.25)** | (0.54, 5.44) |  |  |
| Two or more | 1.3 (1.2) | (-1.01, 3.64) | 1.2 (1.4) | (-1.52, 3.98) | 2.13 (1.36) | (-0.54, 4.80) |  |  |
| vs. None | ref |  | ref |  | ref |  |  |  |
| Analyses involved regressing quality of life domains or psychological outcomes on number of ICD10 medical conditions (one or more vs. none; one, two or more, vs. none) while controlling for sociodemographic information such as gender, ethnicity, marital status, highest education levels, age, age of onset, and psychotic disorder categories; values in bold indicate significance at p<.05. | | | | | | | | |
