# Supplementary material for: Comorbid physical illnesses in adult outpatients with psychotic disorders: risk factors, psychological functioning, and quality of life outcomes
Source: Soc Psychiatry Psychiatr Epidemiol. 2021 Feb 22;56(9):1633–43. doi: 10.1007/s00127-021-02034-8 (PMC8429359; doi:10.1007/s00127-021-02034-8)
Supplement: Supplementary file 2 — Supplementary file2 (DOCX 16 KB) [file 127_2021_2034_MOESM2_ESM.docx]

| Supplementary Table II: Multiple linear regression analyses of all ICD-10 medical conditions, DASS21, and WHOQOL-BREF scores. | | | | | | | | |
| --- | --- | --- | --- | --- | --- | --- | --- | --- |
|  | QUALITY OF LIFE (WHOQOL-BREF) | | | | | | | |
|  | Physical Health | | Psychological Health | | Social Relationships | | Environment | |
| ICD-10 medical conditions | *B (SE)* | 95% CI | *B (SE)* | 95% CI | *B (SE)* | 95% CI | *B (SE)* | 95% CI |
| *One or more* | **-0.72 (0.28)** | (-1.27, -.17) | -0.22 (0.34) | (-.887 .443) | -0.53 (.36) | (-1.243, .179) | -.14 (0.3) | (-.737, .459) |
| One | -0.60 (0.33) | (-1.24, .040) | 0.045 (0.40) | (-0.73, 0.82) | -0.35 (0.42) | (-1.19, 0.48) | -0.02 (0.3) | (-0.72, 0.69) |
| Two or more | **-0.88 (0.36)** | (-1.58 -.180) | -0.57 (0.43) | (-1.42, 0.28) | -0.76 (0.46) | (-1.66, 0.14) | -0.30 (0.4) | (-1.07, 0.47) |
| vs. None | ref |  | ref |  | ref |  | ref |  |
|  | PSYCHOLOGICAL OUTCOME MEASURES (DASS 21) | | | | | |  |  |
|  | Anxiety | | Depression | | Stress | |  |  |
| ICD-10 medical conditions | *B (SE)* | 95% CI | *B (SE)* | 95% CI | *B (SE)* | 95% CI |  |  |
| *One or more* | **2.5 (0.9)** | (0.63, 4.27) | **2.7 (1.1)** | (0.56, 4.87) | **2.61 (1.10)** | (.53, 4.70) |  |  |
| One | **3.3 (1.1)** | (1.19, 5.46) | **3.8 (1.3)** | (1.33, 6.36) | **2.99 (1.25)** | (0.54, 5.44) |  |  |
| Two or more | 1.3 (1.2) | (-1.01, 3.64) | 1.2 (1.4) | (-1.52, 3.98) | 2.13 (1.36) | (-0.54, 4.80) |  |  |
| vs. None | ref |  | ref |  | ref |  |  |  |
| Analyses involved regressing quality of life domains or psychological outcomes on number of ICD10 medical conditions (one or more vs. none; one, two or more, vs. none) while controlling for sociodemographic information such as gender, ethnicity, marital status, highest education levels, age, age of onset, and psychotic disorder categories; values in bold indicate significance at p<.05. | | | | | | | | |
